# Beta-Catenin Signaling Negatively Regulates Intermediate Progenitor Population Numbers in the Developing Cortex

**DOI:** 10.1371/journal.pone.0012376

**Published:** 2010-08-25

**Authors:** Christopher A. Mutch, Jessica D. Schulte, Eric Olson, Anjen Chenn

**Affiliations:** 1 Department of Pathology, Northwestern University Feinberg School of Medicine, Chicago, Illinois, United States of America; 2 Department of Neuroscience and Physiology, State University of New York, Upstate Medical University, Syracuse, New York, United States of America; Universidade Federal do Rio de Janeiro, Brazil

## Abstract

Intermediate progenitor cells constitute a second proliferative cell type in the developing mammalian cerebral cortex. Little is known about the factors that govern the production of intermediate progenitors. Although persistent expression of stabilized β-catenin was found to delay the maturation of radial glial progenitors into intermediate progenitors, the relationship between β-catenin signaling and intermediate progenitors remains poorly understood. Using a transgenic reporter mouse for Axin2, a direct target of Wnt/β-catenin signaling, we observed that β-catenin signaling is decreased in intermediate progenitor cells relative to radial glial progenitors. Conditional deletion of β-catenin from mouse cortical neural progenitors increased intermediate progenitor numbers, while conditional expression of stabilized β-catenin reduced the intermediate progenitor population. Together, these findings provide evidence that β-catenin signaling in radial progenitors negatively regulates intermediate progenitor cell number during cortical development.

## Introduction

During mammalian cortical neurogenesis, neural progenitors proliferate to produce neurons that form the six-layered cerebral cortex. Two distinct populations of progenitor cells produce the excitatory projection neurons that populate the cortical plate. Radial progenitors, also known as radial glia, have a characteristic polarized epithelial morphology with an apical process that abuts the lateral ventricle and a basal process that extends toward the pial surface of the developing cortex [1]–[3]. Radial progenitors can either divide symmetrically to self-renew or divide asymmetrically to produce another radial progenitor and either a neuronal or intermediate progenitor daughter cell [4]–[6].

Intermediate progenitors arise from radial glia [7] and emerge shortly after cortical neurogenesis commences [7], [8]. In contrast to radial progenitors, intermediate progenitors are multipolar and lack ventricular contacts [9]. While radial progenitors divide at the apical surface of the ventricular zone (VZ), intermediate progenitors divide basally (abventricularly), and in the middle and late stages of cortical neurogenesis comprise the subventricular zone (SVZ) of the developing cortex [10].

Although basal mitotic figures in the developing cortex were first described over three decades ago [11], recent advancements in live imaging techniques have led to increased understanding of these divisions [4]–[6], [12]. In contrast to radial progenitors, intermediate progenitors appear to divide symmetrically, producing either pairs of neurons or intermediate progenitors [9], [12]. The resultant increase in neuronal production has been proposed as a mechanism by which cortical complexity may be increased in higher mammals [7], [13]–[15].

The production of intermediate progenitors from radial progenitors appears to require transcription factors important in the stepwise maturation of neurons from dividing progenitors. Recent studies suggest that the T-brain gene-2 (*Tbr2*) transcription factor is both necessary and sufficient for the development of intermediate progenitors in the developing cortex [16]. Conditional ablation of *Tbr2* in the developing cortex led to the loss of intermediate progenitor cells [16], [17]. Additionally, forced expression of *Tbr2* is sufficient to induce intermediate progenitor cell fate in radial progenitor cells [16]. The proneural gene *Ngn2* also regulates the progression of progenitor to postmitotic neuron, but its role in intermediate progenitor development changes during cortical development. Loss of *Ngn2* or misexpression of *Mash1* (repressed by *Ngn2*) early in cortical development leads to an expansion of the basal intermediate progenitor pool at the expense of early neuronal production [18]. In contrast, later in cortical development, overexpression of *Ngn2* promotes increased basal divisions [4]. Later in development, Ngn2 may function to regulate intermediate progenitors via induction of the zinc-finger transcription factor Insm1 [19]. A recent study showed that Insm1 can regulate the conversion of radial progenitors to intermediate progenitors, and deletion of *Insm1* reduces the number of intermediate progenitors, while overexpression increases basal divisions and Tbr2 expression [20].

Together, these studies suggest the possibility that intermediate progenitor production is influenced by factors that regulate radial glial differentiation, and factors that promote differentiation may increase the conversion of radial glia into intermediate progenitors, while factors that delay differentiation would decrease the production of intermediate progenitors. In developing cortex, β-catenin activity promotes progenitor production by increasing cell cycle re-entry [21], while inhibition of β-catenin causes premature cell cycle exit and differentiation [22]. Our previous work showed that transgenic overexpression of a stabilized β-catenin appears to delay the maturation of radial glia into intermediate progenitors, suggesting that downregulation of β-catenin signaling may be important in the development of intermediate progenitors [23]. The studies presented here examine further the relationship between β-catenin signaling levels and intermediate progenitor development.

## Results

### Visualization of β-catenin signaling in the E14.5 cortex

Our prior work suggested that downregulation of β-catenin signaling might be a necessary step in intermediate progenitor development [23]. In order to better characterize where β-catenin signaling is downregulated in the developing cortex, we utilized Axin2-dEGFP reporter transgenic mice [24]. Axin2 is a direct target of Wnt/β-catenin signaling and only expressed in tissues with active Wnt/β-catenin signaling [24]–[26]. The Axin2-d2EGFP transgenic mouse line was constructed by cloning the 5.6 kb fragment of mouse genomic DNA upstream from the translation start site of *Axin2* including the promoter, the first exon, and the first intron of Axin2 [24]. The *Axin2* regulatory sequence drives the expression of destabilized Green Fluorescent Protein (d2EGFP), a GFP variant designed to be rapidly degraded (half-life of ∼2 hours, and the short half-life of d2EGFP facilitates examining reductions in signaling not possible with more stable reporters such as β-galactosidase. GFP expression in these mice matches the expression of endogenous Axin2 [24], and has been used to accurately visualize β-catenin signaling in the brain and other tissues [24], [27], [28].

**Figure 1:**
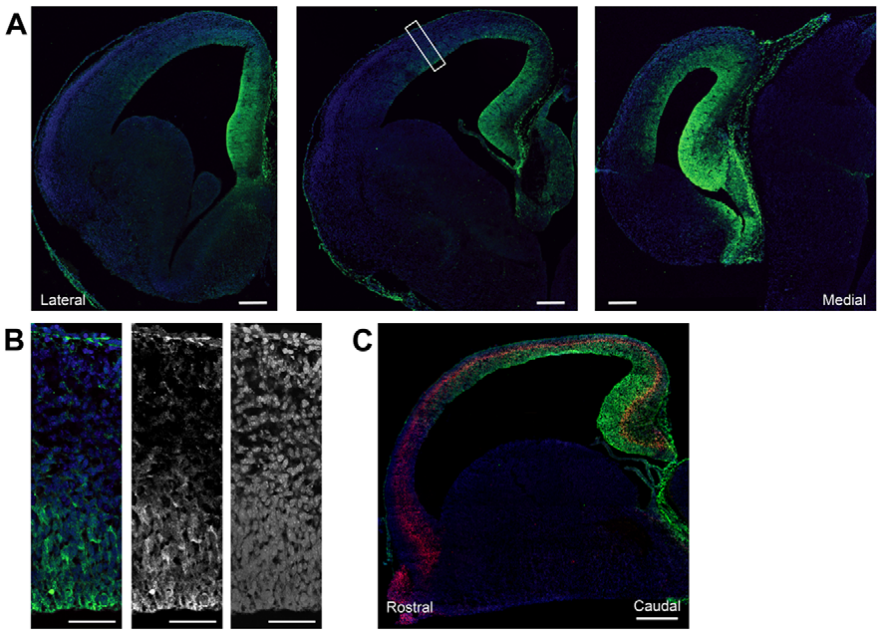
Beta-catenin signaling in the E14.5 cortex. (**A**) This series of images represent rostral, middle, and caudal coronal sections (from left to right) of an E14.5 Axin2-d2EGFP forebrain hemisphere, stained with anti-GFP antibody (green) and DAPI (blue). Images were obtained by overlapping 1 µm confocal optical sections. Signaling is strongest medially and progressively decreases through the dorsal and lateral cortex in all sections. (**B**) The dorsolateral cortex of the middle section shown in A (white box) is shown at higher magnification. In this image, staining with anti-GFP antibody shows Axin2-d2EGFP expression is present in the VZ, while d2EGFP signal in the IZ and CP is reduced. (**C**) A parasagittal section through the dorsolateral cortex of an Axin2-d2EGFP brain shows an Axin2 gradient rostral high to caudal low. The Axin2-d2egfp signal is stronger in the ventricular zone, and reduced in IZ and CP, along the length of the rostro-caudal axis. Section was stained with anti-GFP antibody (green), DAPI (blue), and anti-Tbr2 antibody (red). Tbr2 staining suggests a rostral low to caudal gradient. Scale bars are 200 µm (**A**) 50 µm (**B**) and 500 µm (**C**).

As intermediate progenitors are most prominent during the middle to late neurogenic period [10], we chose to examine β-catenin signaling in the developing E14.5 cortex, approximately the midpoint of cortical neurogenesis. We examined coronal sections of E14.5 Axin2-d2EGFP cortices for d2EGFP fluorescence and found d2EGFP expression localized in the ventricular zone (VZ) cells on the medial, dorsal and lateral aspects of the lateral ventricle (Figure 1A).

Consistent with previous reports using other reporters of Wnt/β-catenin signaling, the level of β-catenin signaling was highest in the medial cortex, reflecting a gradient complementary to the normal developmental gradient of neurogenesis in the developing cortex [29]–[31]. β-catenin signaling was highest in the developing hippocampus and declined in a dorsal cortex high to lateral cortex low gradient (Figure 1A). d2EGFP signal was essentially undetected in the ganglionic eminence and other ventral structures of the telencephalon. When examined at higher magnification in the developing cortex, apical ventricular zone (VZ) cells showed high levels of Axin2-d2EGFP reporter activity (Figure 1B), while outside the VZ, signal appeared reduced in the subventricular zone (SVZ), intermediate zone (IZ), and cortical plate (CP). d2EGFP expression in the sagittal plane of the dorsolateral cortex also suggests a caudal high to rostral low gradient of Axin2, which opposes the medial/caudal low to lateral/rostral high expression gradient of Tbr2, a marker for intermediate progenitors (Figure 1C). Together, these findings suggest that β-catenin signaling is involved with the lateral/rostral to medial/caudal maturation gradient of the cerebral cortex.

### Beta-catenin signaling levels are reduced in intermediate progenitors

In the developing cortex, dividing intermediate progenitors are found primarily in the SVZ [10]. While intermediate progenitor cells express several molecular markers including Cux1, Cux2, Svet1, and Tbr2 [32]–[34], Tbr2 is exclusively expressed in intermediate progenitor cells. Moreover, Tbr2 appears to define intermediate progenitors, as conditional deletion of *Tbr2* results in loss of intermediate progenitors, while overexpression can convert radial glia into intermediate progenitors [16]. Because of these features, the expression of Tbr2 has been used in many studies to identify intermediate progenitor cells [23], [32], [35], [36].

**Figure 2:**
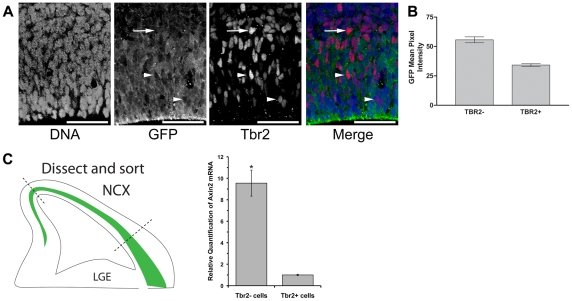
Beta-catenin signaling is decreased in intermediate progenitor cells. (**A**) Cortex from E14.5 Axin2-d2EGFP transgenic mice stained for GFP (green), Tbr2 (red), and DNA (blue). Arrows highlight examples of Tbr2+ cells outside the VZ, while arrowhead point to Tbr2+ cells still in the VZ. Tbr2+ cells have decreased d2EGFP signal in both the VZ and SVZ. (**B**) To quantify differences in signaling between Tbr2+ and Tbr2- cells, fluorescence intensity of Tbr2 and GFP was measured in the area over each of the nuclei in the VZ and SVZ (right). Scale bars are 50 µm. * * = P<0.001, t test, two-tailed. (**C**) To further quantify signaling differences between Tbr2+ and Tbr2- cells, dorsolateral neocortices from E13.5 Eomes::GFP embryos (n = 3) were FACS sorted for GFP+ and GFP- cells (see cartoon). mRNA from these cells was isolated and reverse transcribed into cDNA for real-time PCR analysis. Using comparative C_T_ analysis with Actin as an endogenous control, real-time PCR showed that Axin2 transcript was 9.55±1.21 fold higher in the Tbr2- cells than in the Tbr+ cells. n = 3. Error bars represent SEM, * = p<0.05, paired t-test, two-tailed.

To examine whether the apparent reduction of signaling in the SVZ stems from differences in signaling in intermediate progenitors, we examined GFP levels in Tbr2-expressing cells. In the developing cerebral cortex, *Tbr2* is expressed in high lateral to low medial, and high rostral to low caudal gradients in mouse [37] and human [38]. Upon examining signaling and Tbr2 expression on a cellular level, we found that Tbr2-expressing cells had reduced d2EGFP reporter expression, compared with Tbr2 negative cells in the VZ or SVZ (Figure 2B; N = 3, n = 226 cells total). Furthermore, Tbr2+ cells that had not yet left the VZ also had decreased d2EGFP expression (arrowheads, Figure 2A).

To corroborate these findings, we examined Axin2 transcript levels in intermediate progenitors in developing cortex using real time qRT-PCR. Here, we used a transgenic mouse that contains the coding sequence for EGFP inserted into the mouse bacterial artificial chromosome (BAC) RP23-235G22 at the ATG transcriptional initiation site for the Eomes (Tbr2) gene so that EGFP expression reflects the endogenous expression of Tbr2. GFP-expressing (representing Tbr2-expressing) cells and GFP-negative cells were flow sorted from the dorsal neocortex of E13.5 embryos [39], [40]. Axin2 mRNA levels were 9.55±1.21 fold higher in the GFP- (Tbr2-) cells than in the GFP+ (Tbr+) cells (Figure 2C; n = 3 animals, p<0.05, paired t-test). Together, these findings provide support that reduced levels of β-catenin signaling correlate with intermediate progenitor development.

### Loss of β-catenin increases the intermediate progenitor population

**Figure 3:**
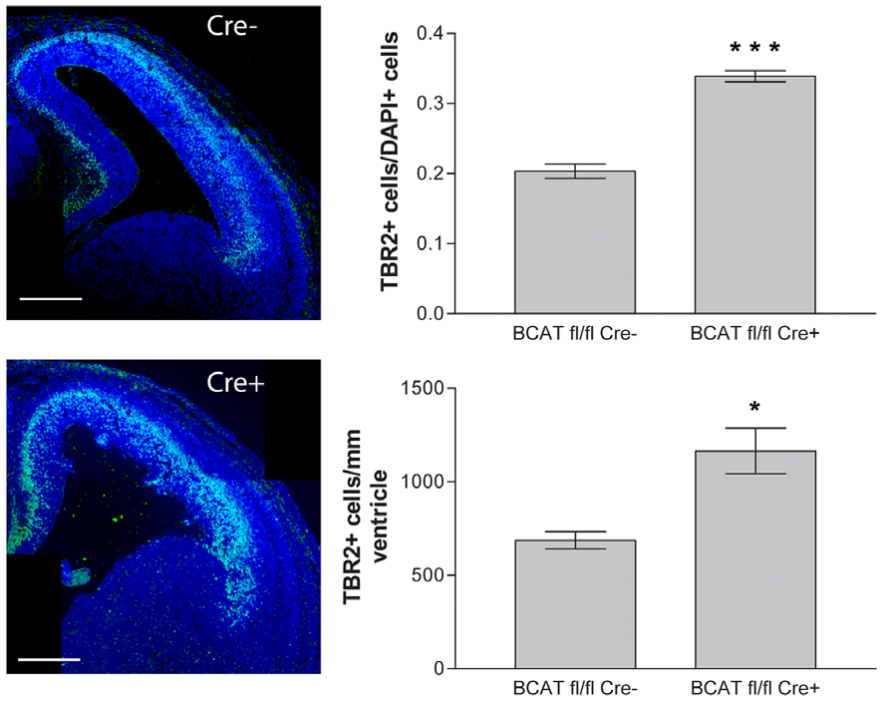
Conditional deletion of β-catenin increases the Tbr2+ intermediate progenitor population. Equivalent coronal sections of E14.5 β-catenin^fl/fl^/Nes-Cre+ and β-catenin^fl/fl^/Nes-Cre- cortices were stained for Tbr2 and DAPI (pseudocolored green (Tbr2) and blue (DAPI) in merged image). The fraction of total cells expressing Tbr2 was significantly increased in β-cat^fl/fl^/Nes-Cre+ cortices when compared to control (top graph). The number of Tbr2-expressing cells per unit ventricular surface length was also increased compared with control (bottom graph). Scale bars are 250 µm. * = P<0.05, t test, two-tailed; * * * = P<0.001, t test, two-tailed.

**Figure 4:**
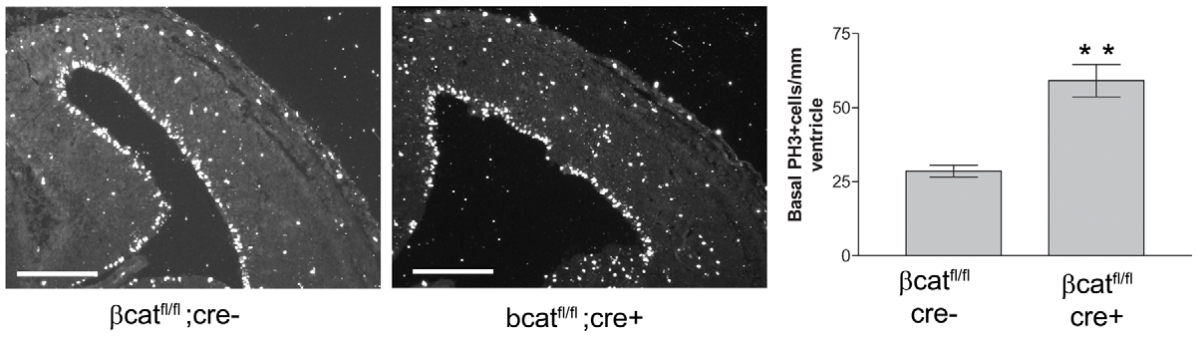
Conditional deletion of β-catenin increases the occurrence of basal mitoses. Staining for PH3 reveals mitotic cells in control βcat^fl/fl^/cre- and βcat^fl/fl^/cre+ cortex. Basal mitoses per mm of ventricular surface were increased in βcat^fl/fl^/cre+ cortices. Arrowheads highlight examples of basal PH3+ cells. Scale bars are 250 µm. * * = P<0.01, t test, two-tailed.

To investigate whether downregulation of β-catenin is sufficient to induce the production of intermediate progenitors, we crossed β-catenin^lox(ex2–6)^ mice, in which exons 2 (which contains the ATG translational start) to 6 of β-catenin are flanked by LoxP sites [41] with Nes-Cre mice, which exhibit widespread Cre recombinase in neural progenitor cells by E11 [42]. Upon cre-mediated recombination, the β-catenin floxed allele is converted into a floxdel allele unable to generate a functional β-catenin protein [41]. We examined the number of Tbr2-expressing cells in equivalent sections of both β-catenin^fl/fl^/Nes-Cre+ (β-cat^fl/fl^/Cre+) and β-catenin^fl/fl^/Nes-Cre- (β-cat^fl/fl^/Cre-) cortices (Figure 3). Deletion of β-catenin alleles in β-cat^fl/fl^/Cre+ cortices (Figure 3) led to a significant increase (1.7 fold, P = 0.0005) in the proportion of cortical cells expressing Tbr2 (33.9%±1.4% in β-cat^fl/fl^/Cre+ sections, N = 3, n = 9720 cells; 20.3%±1.0% in β-cat^fl/fl^/Cre- sections, N = 3, n = 8985 cells). To assess the numbers of basal-dividing intermediate progenitors, we stained β-cat^fl/fl^/Cre+ and β-cat^fl/fl^/Cre- cortical sections for the phosphorylated form of histone H3 (PH3) (Figure 4). Loss of β-catenin increased the number of basal PH3+ cells (59.2±5.4 cells/mm ventricular surface, N = 3, n = 94 cells in β-cat^fl/fl^/Cre+ vs. 28.7±2.0 cells/mm, N = 3, n = 59 cells in β-cat^fl/fl^/Cre-; P = 0.0062), whereas mitotic apical progenitors showed no change in density (103.7±11.4 cells/mm ventricular surface in β-cat^fl/fl^/Cre+ (N = 3, n = 165 cells) vs. 104.3±5.3 cells/mm, in β-cat^fl/fl^/Cre- (N = 3, n = 179 cells); P = 0.9627, data not shown).

To examine whether downregulating β-catenin signaling specifically may regulate the transition from radial glial cells to intermediate progenitors, we electroporated E13.5 WT brains with a DNA construct driving expression of an N-terminal truncated TCF4. This form of TCF4 lacks the binding site for β-catenin and functions as a dominant negative by competing with endogenous TCF/LEF members for promoter [22], [43], [44]. Electroporation with DNTCF4 caused a decrease in the fraction of Pax6-expressing cells (0.173±0.006; N = 3, n = 242 cells) compared to electroporation with GFP (0.339±0.031; N = 3, n = 579 cells) (Figure S1). Taken together, these results indicate that the deletion of β-catenin increases the generation of intermediate progenitors.

### Regional alterations in cortical TBR2 expression

**Figure 5:**
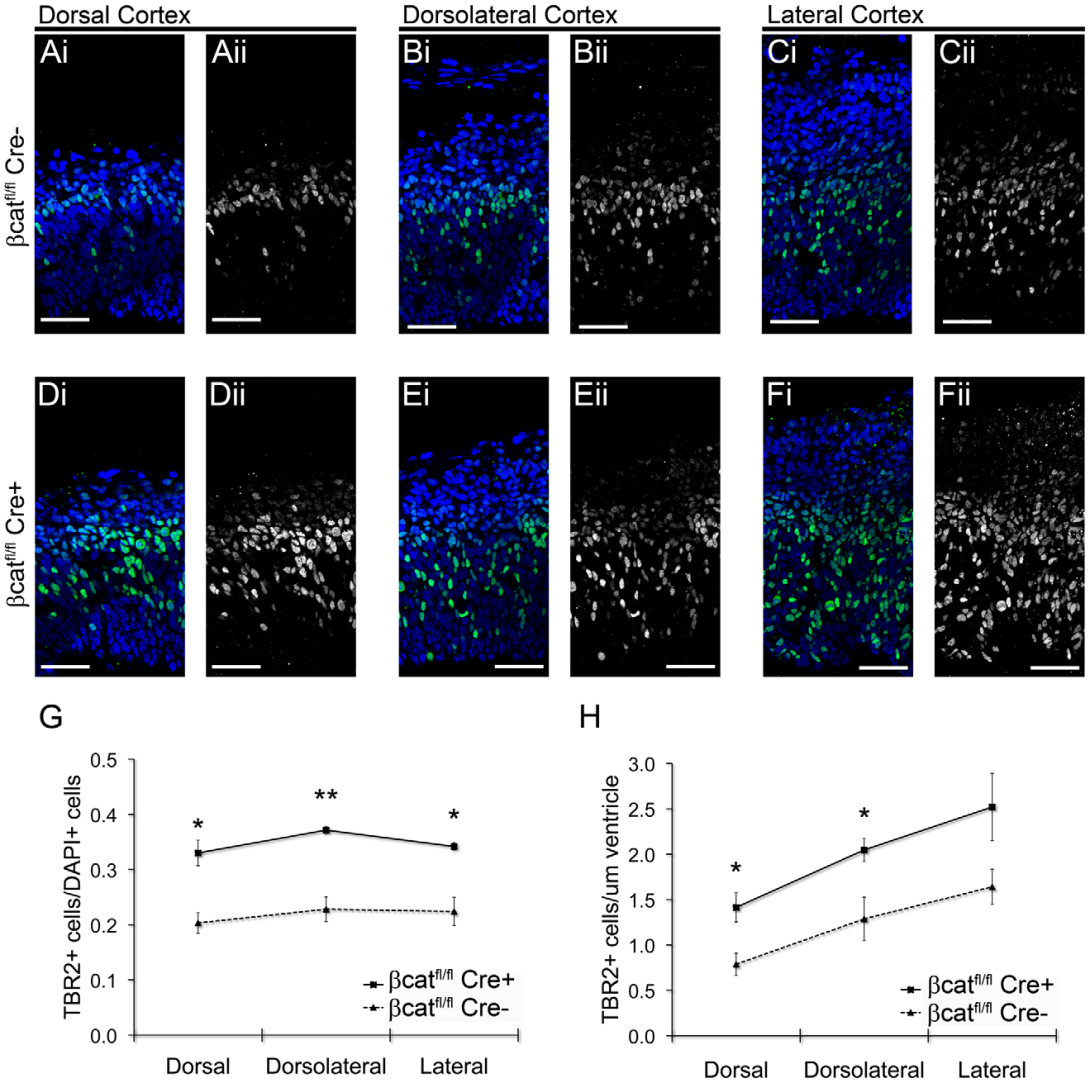
Regional differences in Tbr2 expression. Equivalent coronal sections of E14.5 control β-catenin^fl/fl^/Nes-Cre- and conditional knock out β-catenin^fl/fl^/Nes-Cre+ cortices were stained with the intermediate progenitor marker Tbr2 and DAPI. Merged images are shown in panels **A,B**, etc.; Tbr2 alone is shown in **Ai, Bi** etc. High resolution confocal images were taken from the extreme dorsal (**A,D**) and extreme lateral (adjacent to the LGE) (**C,F**) aspects of each cortex. Additional dorsolateral (**B,E**) regions were imaged at the midpoint between the dorsal and lateral regions. In each region, a greater proportion of cells expressed Tbr2 in β-cat^fl/fl^/Nes-Cre+ sections when compared to control β-cat^fl/fl^/Nes-Cre- (**G**). Within both β-cat^fl/fl^/Nes-Cre+ and Cre- sections, the proportion of Tbr2+ cells to total cells was consistent across the three regions (**G**). Although the size of the Tbr2+ SVZ increased progressively from dorsal sections to lateral sections in both β-catenin phenotypes, this increase was offset by concurrent increases in CP size. When Tbr2 expression was normalized by ventricular surface length (**H**), the increased intermediate progenitor population in the more lateral regions of both groups became apparent. Scale bars are 50 µm. * * = P<0.01, * = P<0.05, t test, two-tailed.

Our initial studies support previous observations that a Tbr2 expression gradient might parallel the dorsal to ventral developmental gradient in the cortex (Figure 3) [31]. To compare regional differences in Tbr2 expression following deletion of β-catenin, we examined the most dorsal aspect of the cortex (Figure 5A,D; N = 3, n = 1223 cells), the lateral cortex adjacent to the ganglionic eminence (Figure 5C,F; N = 3, n = 1660 cells), and the dorsolateral cortex, equidistant between the dorsal and lateral subsections (Figure 5B,E; N = 3, n = 2202 cells). Deletion of β-catenin (β-cat^fl/fl^/Cre+) significantly increased the fraction of total cells expressing Tbr2 in all three regions (Figure 5G). Within each group the total fraction of dorsal, dorsolateral and lateral cortical cells expressing Tbr2 were consistent (Table 1). However, when we calculated the number of Tbr2 expressing cells for eachµm surface length of the ventricle, regional changes in expression became apparent (Figure 5H, Table 1). In β-cat^fl/fl^/Cre- cortices, there were more than twice as many Tbr2+ cells perµm in the lateral cortex when compared to the dorsal cortex. In β-catenin β-cat^fl/fl^/Cre+ cortices we observed a slightly smaller gradient (∼1.7 fold increase) between dorsal and lateral cortex Tbr2 expression (Figure 5H, Table 1).

**Table 1:** Regional changes in cortical Tbr2 expression.

	Tbr2+ cells/DAPI+ cells			Tbr2+ cells/µm ventricle		
Cortical Region	β-catenin^fl/fl^Nes-Cre-	β-catenin^fl/fl^Nes-Cre+	P Value(t test)	β-catenin^fl/fl^Nes-Cre-	β-catenin^fl/fl^Nes-Cre+	P value(t test)
**Dorsal**	0.203±0.019	0.330±0.023	P = 0.013	0.411±0.067	0.739±0.085	P = 0.037
**Dorsolateral**	0.228±0.022	0.371±0.006	P = 0.004	0.673±0.126	1.070±0.067	P = 0.049
**Lateral**	0.224±0.026	0.342±0.007	P = 0.011	0.858±0.102	1.318±0.194	P = 0.103

All values are ± s.e.m. P values were calculated using Student's t test (two-tailed).

### Stabilization of β-catenin decreases intermediate progenitor population levels

Because loss of β-catenin appears to increase the occurrence of basal intermediate progenitors, we investigated whether increasing β-catenin could reduce intermediate progenitor numbers. The N-terminal portion of β-catenin containing GSK3β phosphorylation sites required for its degradation are encoded by exon 3 of the β-catenin locus, and removal of this region results in the production of a stabilized protein [45]. To examine the effects of increasing β-catenin, we crossed mice with loxP sites flanking the third exon of β-catenin (Ctnnb1^lox(ex3)^) [46] with Nestin cre mice.

**Figure 6:**
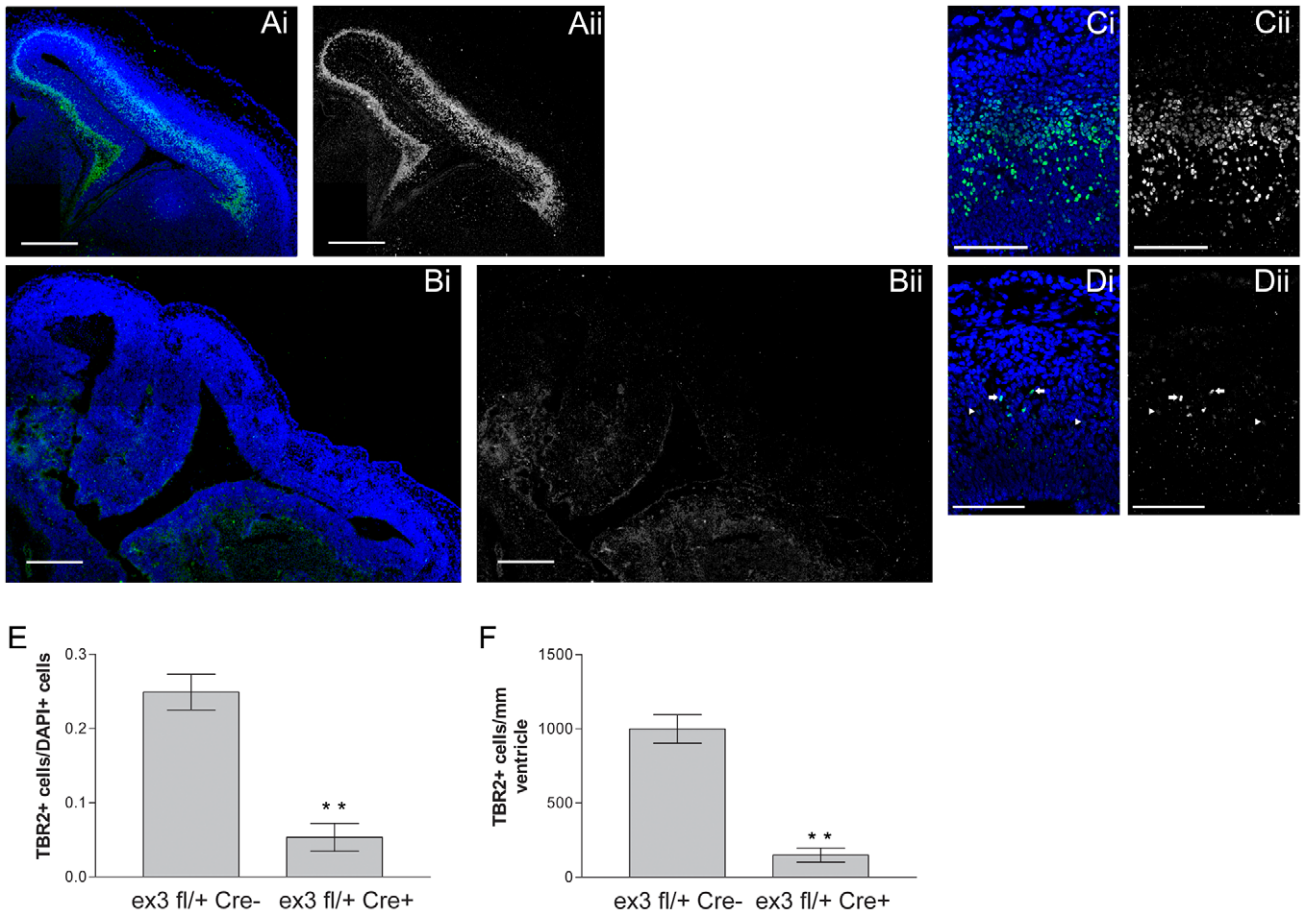
Conditional stabilization of β-catenin decreases frequency of Tbr2-expressing cells. Coronal sections of E14.5 control and β-catenin ΔEx3 cortices were stained with the intermediate progenitor marker Tbr2 and DAPI. Merged images are shown in panels **Ai,Bi**, etc.; Tbr2 alone is shown in **Aii,Bii**, etc. The proportion of total cells expressing Tbr2 was significantly decreased in β-catenin ΔEx3 cortices (**E**) when compared to the NesCre- control. To control for the possibility of changes in total cell number between β-catenin ΔEx3 and control cortical sections, we also normalized the density of Tbr2+ cells by the length of the ventricular surface in each cortex. This analysis also resulted in a markedly decreased Tbr2+ intermediate progenitor population in cortices (**F**) when compared to NesCre- control. The bottom panels show greater detail of β-catenin ΔEx3 (**Di,Dii**) and control (**Ci,Cii**) cortical sections. While some cells showed strong expression of Tbr2 (**Dii**, arrows) similar to control (**Cii**), the majority of Tbr2+ cells in ΔEx3 cortices weakly expressed Tbr2 (**Dii**, arrowheads show examples). Scale bars are 250 µm in low power panels, 100 µm in high power panels. * * = P<0.01, t test, two-tailed.

**Figure 7:**
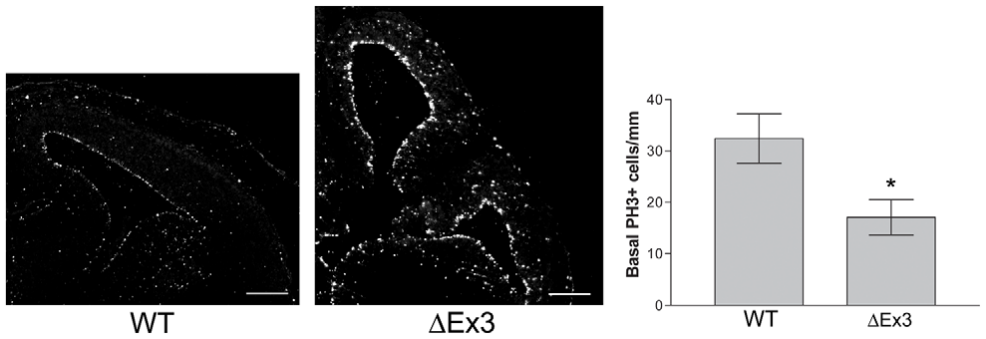
Conditional stabilization of β-catenin decreases frequency of basal mitoses. Staining for PH3 reveals mitotic cells in control (**A**) and ΔEx3 transgenic (**B**) cortex. Tbr2 channel alone is shown in **Ai, Bi** etc. Basal mitoses per mm of ventricular surface were decreased in β-catenin ΔEx3 transgenic cortices (**C**). Arrowheads highlight examples of basal PH3+ cells. Scale bars are 250 µm. * = P<0.05, t test, two-tailed.

E14.5 cortices from Catnb ^lox(Ex3)^/+; NesCre (hereafter referred to as ΔEx3 transgenic) exhibited large expansion in ventricular surface area compared with control mice (Figure 6B, Table S1) as reported previously [21], [23], [47]. Examination of Tbr2 expression in the dorsolateral cortex (Figure 6C,D) showed that not only do fewer cells express Tbr2 in ΔEx3 transgenic cortex, but Tbr2-expressing cells express it weakly (arrowheads, Figure 6D). While there were some cells that strongly express Tbr2 (arrows, Figure 6D,E), these cells were far less frequent compared with Nes-Cre- control brains (Figure 6C). Moreover, the fraction of total cortical cells expressing Tbr2 was dramatically reduced in ΔEx3 transgenic cortices (0.0534±0.0188 (N = 3, n = 26535 cells) vs. 0.249±0.024 (N = 3, n = 15509 cells) in Nes-Cre-, a 4.7 fold decrease (P = 0.0031, t-test)). We also found that frequency of basally located PH3+ cells was significantly decreased (1.9 fold, P = 0.0258, t-test) in ΔEx3 transgenic cortices (17.08±4.43 cells/mm; N = 6, n = 1135) compared with Nes-Cre- cortices (32.43±4.80 cells/mm; N = 5, n = 3852 cells) (Figure 7). Apical progenitors showed did not show a significant change in density (75.3±8.2 cells/mm ventricular surface, in ΔEx3 cortices (N = 3, n = 521 cells) vs. 106.6±13.6 cells/mm in Nes-Cre- control cortices (N = 3, n = 152 cells); P = 0.1229; data not shown).

To complement these studies, we examined expression of Pax6 following *in utero* electroporation and incubation of Δ90β-catenin, a stabilized form of β-catenin that leads to overactivation of signaling [21]. Electroporation with Δ90β-catenin-GFP increased the fraction of Pax6-expressing cells (0.534±0.015; N = 3, n = 242 cells) compared to electroporation with GFP (0.339±0.031; N = 3, n = 579 cells) (Figure S1). Together with the loss-of-function studies, these gain-of-function studies suggest that β-catenin levels can regulate the number of intermediate progenitors in the developing cortex.

## Discussion

Using a transgenic mouse reporter of canonical Wnt/β-catenin signaling, we observed that signaling in cortical VZ progenitors is reduced in the SVZ, and cells expressing the intermediate progenitor marker Tbr2 have lower levels of β-catenin signaling than their Tbr2 negative neighbors. Real-time RT-PCR experiments measuring Axin2 transcript levels in the GFP+ cells of Eomes::GFP mice provide further evidence that β-catenin signaling is reduced in intermediate progenitors. Conditional deletion of β-catenin leads to increased numbers of intermediate progenitors, while conditional expression of stabilized β-catenin reduces numbers of intermediate progenitors. Together, these findings suggest β-catenin signaling is a negative regulator of intermediate progenitor cell production.

How conclusive is the evidence for downregulation of β-catenin signaling in intermediate progenitors? Reductions in Wnt/β-catenin signaling are difficult to visualize with most reporter systems because of reporter perdurance (e.g. β-galactosidase expression). While expression of Wnt/β-catenin target genes can reveal reductions in signal, using *in situ* hybridization to obtain single cell resolution while examining expression of other proteins is not feasible in the closely packed cellular environment of the developing cortex. Instead, to visualize downregulation of signaling in developing cortex, we utilize a destabilized EGFP controlled by *Axin2* regulatory sequences as a reporter for Wnt signaling. Our observations that Axin2-regulated dGFP signal is reduced outside of the VZ support our prior findings [22] using a reporter construct that expresses dGFP under the control of another well-characterized β-catenin responsive promoter (TOPdGFP) [48]. Furthermore, by examining Tbr2-expressing vs. Tbr2-negative cells purified from embryonic cortex we found that Axin2 message is dramatically reduced in Tbr2-expressing cells. Unlike many Wnt/β-catenin target genes, which appear to be tissue-specific, *Axin2* is expressed in most if not all sites of signaling, and is believed to be a universal indicator of Wnt/β-catenin activity [24], [49]. Although crosstalk from other pathways could potentially act on the *Axin2* promoter, our observations are consistent with other published reports of both *Axin2* expression and other Wnt/β-catenin signaling reporters in the cortex [24], [29]–[31], [50].

Prior studies have shown that radial progenitors can generate cortical neurons directly or indirectly via production of intermediate progenitors [6], [12]. Our observations that loss of β-catenin appears to cause radial glial progenitors to prematurely differentiate into intermediate progenitors support the results from two recent studies where forced expression of *Tbr2* [16] or *Insm1* [20] in VZ progenitors increases conversion of VZ progenitors into intermediate progenitors. In contrast, deletion of other genes known to maintain radial progenitors such as *Foxg1* and *Pax6* has resulted in a corresponding decreased number of intermediate progenitors and reduced expression of *Tbr2* [36], [51], [52]. Together, these results suggest that maintaining radial glial identity and determination of the fate of their daughters may be distinctly regulated, and distinct pathways may control whether a radial glial cell differentiates into an intermediate progenitor or a postmitotic cortical neuron.

As the fraction of intermediate progenitor divisions that generate additional intermediate progenitors (proliferative) appears to decline steadily during cortical development [12], it is likely that intermediate progenitor number is largely regulated via production from VZ progenitors. Previous observations that basal progenitor numbers increased steadily during cortical development [8] was supported by a recent study examining both the frequency of basal divisions and Tbr2+ expression [12]. Based on the distribution of Tbr2 expressing cells in the developing cortex, the observation that Tbr2 expression follows the known developmental gradient (rostral-lateral first to caudo-medial last) is consistent with the possibility that early VZ progenitors are less likely to generate intermediate progenitors and more likely to produce additional radial progenitors. Because β-catenin signaling appears to be opposing the developmental gradient (medial high to lateral low, caudal high to rostral low), β-catenin signaling in VZ progenitors may influence intermediate progenitor number by delaying VZ maturation during development.

Moreover, our recent observations that the degree of β-catenin signaling declines in the VZ from mid to late cortical development provide further support for the notion that higher β-catenin signaling inhibits intermediate progenitor production [53]. Despite the attractiveness of this model, the fraction of progenitor divisions that generate intermediate progenitors vs. postmitotic cortical neurons during development remains poorly understood, and live imaging is likely necessary to clarify the patterns of cell division that lead to the production of intermediate progenitors vs. neurons.

Although our study suggests that β-catenin reduces the number of intermediate progenitors, a recent study suggests that β-catenin promotes intermediate progenitor production [54]. Retroviral expression of stabilized β-catenin into midgestation cortical progenitors *increased* Tbr2 cell numbers. The differences in timing between our two studies may provide additional insights. Kuwahara et al. utilized retroviral infection of stabilized β-catenin at E12.5, while our approach used Nestin Cre mice to produce stabilized β-catenin beginning at E10.5. In fact, the differences in outcomes support findings from their group suggesting that β-catenin signaling has distinct functions early vs. late in cortical development, with β-catenin promoting self-renewal at E10.5 vs. differentiation at later stages [55]. Further studies using temporally regulated inducible mouse models may provide further insight onto the developmental differences in response to β-catenin signaling.

Inquiry into the interactions between β-catenin and other signaling pathways will likely yield further insight into the regulation of intermediate progenitor cells. Several transcription factors such as *Foxg1* and *COUP-TFI* are expressed in low medial to high lateral gradients that could potentially oppose β-catenin signaling [56], [57]. As discussed above, *Foxg1* haploinsufficiency has been shown to decrease the population of intermediate progenitor cells [52]. It has also been shown that *COUP-TFI* overexpression increased intermediate progenitors, while the loss of *COUP-TFI* decreased the intermediate progenitor population [58]. Intriguingly, COUP-TFI was also shown to repress the β-catenin, Mapk/Erk, and PI3K/Akt signaling pathways, providing a potential mechanism for its patterning and cell fate effects [58].

Recent studies support the importance of other β-catenin interactions in the developing cortex. N-cadherin associates with β-catenin at the apical end feet of radial glial cells, mediating cell-cell adhesion and polarity. The observation that N-cadherin plays an important role in regulating β-catenin signaling in the developing cortex [59] provides a plausible mechanism that may underlie recent studies implicating regulators of radial glial polarity such as MARCKS and APC in radial glial differentiation. Alterations in these and other regulators of cell polarity may ultimately regulate radial glial fate by altering the intracellular localization and signaling of β-catenin, as well as that of N-cadherin [60]–[63]. Future studies will examine whether the adhesive role of β-catenin also contributes to regulation of intermediate progenitors.

It is also important to note that β-catenin has been shown to interact with non-TCF transcription factors such as the SOX family [64]. Sox genes have been shown to play important roles in the developing cortex, including their role in modulating β-catenin signaling [65], [66]. Most Sox factors are involved in the suppression of β-catenin/TCF-4 mediated signaling, while a few factors, such as Sox4, actually enhance β-catenin/TCF-4 signaling [67]. In endoderm formation, β-catenin interacts with Sox17 independently of TCF-4 to activate endoderm genes [68] and in breast cancer, β-catenin and Sox2 interact independently of TCF-4 to activate Cyclin D1 [69]. While β-catenin-Sox interactions regulate many aspects of development, our findings that DN-TCF electroporation reduces the Pax6+ fraction of cortical cells (Figure S1) supports the role of canonical β-catenin/TCF targets in intermediate progenitor fate.

While the genetic approaches used to conditionally activate or delete β-catenin provide insights onto the role of β-catenin in intermediate progenitor development, further studies are required to determine whether β-catenin controls intermediate progenitor development through direct regulation of genes such as *Tbr2* or *Insm1.*

## Materials and Methods

### Animals

All animals were treated according to protocols reviewed and approved by the animal care and use committee at Northwestern University, Animal Study Protocol # 2006-0131, approved by the Northwestern University Office for the Protection of Research Subjects Institutional Animal Care and Use Committee. Beta-catenin^lox(ex2–6)^ mice (B6.129-Ctnnb1<tm2Kem>/KnwJ) [41] and Nes-Cre mice (B6.Cg-Tg(Nes-cre)1Kln/J) [42] were obtained from The Jackson Laboratory (Bar Harbor, ME). Mice were genotyped according to The Jackson Laboratory protocols available at http://jaxmice.jax.org/pub-cgi/protocols/protocols.sh?objtype=protocol&protocol_id=471 (β-catenin^lox(ex2–6)^) and http://jaxmice.jax.org/pub-cgi/protocols/protocols.sh?objtype=protocol&protocol_id=288 (Cre). Axin2-d2EGFP mice [24] were a gift from F. Costantini (Columbia University Medical Center, New York, New York). Beta-catenin^lox(ex3)^ mice were a gift from M. Taketo (Kyoto University, Kyoto, Japan). The construction and genotyping protocols for these mice have been described [46].

E13.5 Eomes::GFP embryos (The Gene Expression Nervous System Atlas Project, NINDS Contract # N01NS02331 to The Rockefeller University, New York, NY) were mated to C57Bl6 (Taconic Labs, Germantown, NY) to generate embryos for flow cytometric sorting of intermediate progenitors (see below).

### Immunofluorescence and Antibodies

Embryonic brain samples were fixed overnight in 4% paraformaldehyde (PFA). Samples were then prepared for sectioning by either vibratome or cryostat. Samples that were to be sectioned by vibratome (Axin2-d2EGFP), were removed from fix and embedded in a 1.5% solution of agarose is phosphate buffered saline (PBS). Once the agarose polymerized, samples were post-fixed in 4% PFA for an additional 30 minutes, mounted, and vibratome sectioned in the coronal plane at 100 µm. Samples to be cryosectioned were transferred to a 30% sucrose solution after fixation for ∼24 hours and then embedded and flash frozen in O.T.C. compound (Tissue-Tek, Torrance, CA). Samples were sectioned in the coronal plane at a thickness of 12 µm and stored at −80°C until use. Because of extreme disruption of the neocortical architecture in the Cre+ Beta-catenin^lox(ex3)^ mice, all coronal images here represent sections taken through the midsection of the eye as a standard landmark.

Primary antibodies used were GFP (rabbit polyclonal, 1∶1000, Invitrogen, Carlsbad, CA; chicken polyclonal, 1∶1000, Abcam, Cambridge, MA), Tbr2 (rabbit polyclonal, 1∶250, Abcam) and Phospho-Histone H3 (Ser10) (rabbit polyclonal, 1∶200, Millipore, Billerica, MA). Secondary antibodies (goat anti-rabbit and goat anti-chicken, 1∶1000) and DAPI (1∶1000) nucleic acid dye were purchased from Invitrogen.

For immunofluorescence, vibratome sections were incubated for 12 h in blocking buffer (5% goat serum and 0.3% Triton X in PBS) at 4°C, followed by incubation for 24 hours in primary antibody diluted with blocking buffer at 4°C. After washing in PBS, sections were incubated with secondary antibody and DAPI diluted in PBS for 12 hours at 4°C.

For cryosections, slides were incubated for 30 minutes in blocking buffer (same as above) at room temperature, and then incubated with primary antibody solution for 16 hours at 4°C, followed by incubation with secondary antibody and DAPI for 2 hours at room temperature. To maintain consistent rostral-caudal positioning, coronal sections through the plane of the embryonic eye structures were used for all analysis.

### Image Acquisition and Analysis

Images used for analysis were acquired with a Zeiss (Oberkochen, Germany) LSM510 confocal microscope in 1 µm optical sections. Identical settings were used to acquire control and experimental images for all experiments. Composites of overlapping high power images were assembled using Photoshop CS4 (Adobe Systems Incorporated, San Jose, CA). Cell counts were compiled using ImageJ (http://rsbweb.nih.gov/ij/). Linear ventricular measurements were calculated by measuring the distance along the ventricular surface of the cortex from the point at which the dorsal and medial walls of the lateral ventricle intersect to the lateral ganglionic eminence. One section per brain was counted/measured. Distances were measured using Metatmorph version 6.3r1 (MDS Analytical Technologies, Toronto, Canada). Lower power images used in some figures were collected with a Nikon (San Diego, CA) TE2000-U inverted fluorescence microscope and Metamorph acquisition software.

### Measuring Fluorescence Intensity in Reporter Mice

Embryonic Day (E)14.5 Axin2-d2EGFP mice were sacrificed and vibratome sections were prepared and co-immunostained with anti-GFP, anti-Tbr2 and DAPI as described above. One micron optical section images were obtained on a Zeiss confocal microscope, with each of the 3 above markers in a separate channel. All sections used were stained together and imaged in the same session with the same settings to minimize intersample variance. Using Metamorph image analysis software, each individual cell in the VZ and SVZ was identified by nuclear DAPI expression. Using the software, a region was created over each cell's nucleus. These regions were saved and transferred to the images containing the Tbr2 image and the d2EGFP image. Again using Metamorph, the mean pixel intensity for both the Tbr2 fluorescence and the corresponding d2EGFP fluorescence of each cell was calculated. This data was then used to compute the difference in fluorescence intensity between Tbr2+ and Tbr2- cells.

### Tissue dissociation and cell sorting

Dorsal neocortices from E13.5 Eomes::GFP embryos were rapidly dissected, meninges removed, then dissociated in 1% trypsin/EDTA (Gibco) for subsequent fluorescence activated cell sorting. Cortices from two GFP+ embryos were used per sorted sample and 3 samples were generated per developmental time point. Cells were sorted into GFP+ and GFP- populations on a Becton Dickinson FACS Vantage Flow Cytometer Cell Sorter (SUNY Upstate Medical Flow Cytometry Unit). Cells were sorted directly into collection tubes containing RNALater™ (Qiagen) to minimize post-sort RNA degradation.

### RNA isolation and quality assessment

High quality total RNA was isolated using the RNAeasy™ (Qiagen) kit and flash frozen. Oneµl of RNA was run on an Agilent 2100 Bioanalyzer using the RNA PicoChip to assess RNA structural quality and quantity. Amplified and terminal-labeled cDNAs were then generated by the WT Sense Target Labeling Protocol (Affymetrix).

### Real-time PCR

Comparative C_T_ analysis was run in singleplex using a StepOne Plus Real-time PCR system (Applied Biosystems). FAM-conjugated Taqman primers for mouse Axin2 or mouse Actin (Applied Biosystems) were mixed with Taqman Master mix, H_2_O, and cDNA made from the FACS-sorted cells isolated from the E13.5 Eomes::GFP brains. Identical volumes of cDNA were loaded for all samples, and samples were run in duplicate. The C_T_ (cycle number at which fluorescence is detected above threshold) was determined by the StepOne software algorithm. ΔC_T_ was obtained by subtracting the mean C_T_ value of the endogenous target (Actin) from the mean C_T_ of the experimental target (Axin2). ΔΔC_T_ was calculated by setting a choosing a baseline sample (ΔΔC_T_ set to 0) and comparing all other samples to this baseline. Results are reported in the form of relative quantification, or RQ = 2∧(- ΔΔC_T_). RQ values were averaged for cDNA from three different Tbr2-egfp brains, with one RQ average for GFP positive-sorted (Tbr2+) cells and one RQ average for GFP negative-sorted (Tbr2-) cells (6 samples analyzed total). Error bars represent SEM, and student's t-test was used to calculate significance.

### Statistical Analysis

Data was compiled using Microsoft Excel 2008 (Microsoft, Redmond, WA). All statistics were computed using Prism 3.0 software (GraphPad, San Diego, CA).

## Supporting Information

Figure S1 Beta-catenin signaling regulates VZ precursors E13.5 embryos were electroporated with (A) Δ 90β-catenin-GFP (N = 3), (B) DNTCF4-GFP (N = 3), or (C) GFP control (N = 3). After 30 hours embryos were sacrificed and stained with antibodies raised against GFP and PAX6, a marker for VZ precursors. The fraction of GFP expressing cells that were also PAX6+ for each group was graphed (D). The fraction of PAX6+ electroporated cells in the three experimental groups was significantly different (p<0.0001, ANOVA; Newman-Keuls post-test analysis: Δ 90β-catenin-GFP vs. DNTCF4-GFP p<0.001, Δ 90β-catenin-GFP vs. GFP p<0.001, DNTCF4-GFP vs. GFP p<0.01). Increased β-catenin signaling by Δ 90β-catenin-GFP increased the fraction of cells that retained PAX6 positivity (0.534±0.015 ) while blocking β-catenin signaling decreased the fraction (0.173±0.006), when compared to control (0.339±0.031). Scale bars are 50 µm. n = 3.
(0.84 MB TIF)

Table S1 Summary of embryos and cell counts. cKO refers to beta-catenin fl/fl Nes-cre+ mice; GOF refers to beta-catenin ex3fl/+ Nes-Cre+ mice.
(0.03 MB DOC)
