# Supplementary figures and images for: Beta-Catenin Signaling Negatively Regulates Intermediate Progenitor Population Numbers in the Developing Cortex
Source: PLoS One. 2010 Aug 25;5(8):e12376. doi: 10.1371/journal.pone.0012376 (PMC2928265; doi:10.1371/journal.pone.0012376)

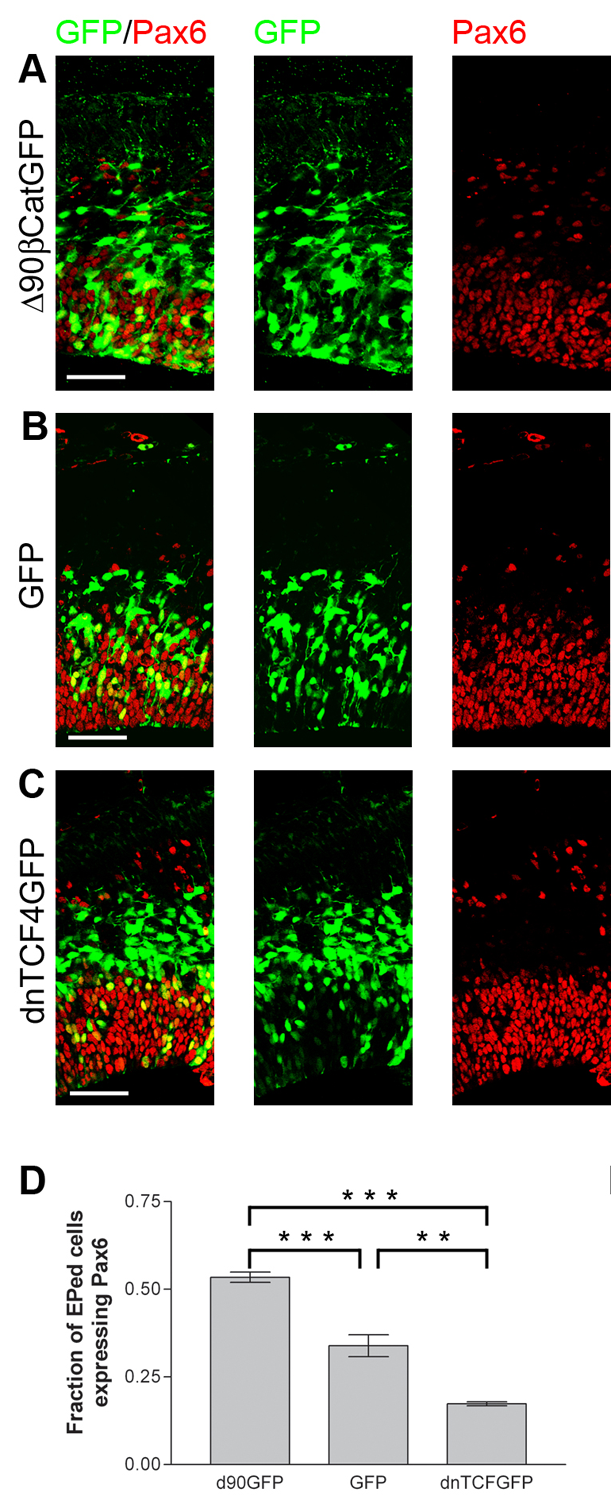

Supplement: Figure S1 — Beta-catenin signaling regulates VZ precursors E13.5 embryos were electroporated with (A) Δ 90β-catenin-GFP (N = 3), (B) DNTCF4-GFP (N = 3), or (C) GFP control (N = 3). After 30 hours embryos were sacrificed and stained with antibodies raised against GFP and PAX6, a marker for VZ precursors. The fraction of GFP expressing cells that were also PAX6+ for each group was graphed (D). The fraction of PAX6+ electroporated cells in the three experimental groups was significantly different (p<0.0001, ANOVA; Newman-Keuls post-test analysis: Δ 90β-catenin-GFP vs. DNTCF4-GFP p<0.001, Δ 90β-catenin-GFP vs. GFP p<0.001, DNTCF4-GFP vs. GFP p<0.01). Increased β-catenin signaling by Δ 90β-catenin-GFP increased the fraction of cells that retained PAX6 positivity (0.534±0.015 ) while blocking β-catenin signaling decreased the fraction (0.173±0.006), when compared to control (0.339±0.031). Scale bars are 50 µm. n = 3. (0.84 MB TIF) [file pone.0012376.s001.tif]
