# Supplementary material for: Beta-Catenin Signaling Negatively Regulates Intermediate Progenitor Population Numbers in the Developing Cortex
Source: PLoS One. 2010 Aug 25;5(8):e12376. doi: 10.1371/journal.pone.0012376 (PMC2928265; doi:10.1371/journal.pone.0012376)
Supplement: Table S1 — Summary of embryos and cell counts. cKO refers to beta-catenin fl/fl Nes-cre+ mice; GOF refers to beta-catenin ex3fl/+ Nes-Cre+ mice. (0.03 MB DOC) [file pone.0012376.s002.doc]

| **Experiment** | **β-catenin cKO** | | **β-catenin GOF** | |
| --- | --- | --- | --- | --- |
| **Strain/**  **Genotype** | **β-catenin^fl/fl^**  **Nes-cre-** | **β-catenin^fl/fl^**  **Nes-cre+** | **β-catenin ex3^fl/+^**  **Nes-Cre-** | **β-catenin ex3^fl/+^**  **Nes-Cre+** |
| **Embryos analyzed** | N=3 | N=3 | N=3 | N=3 |
| **Total cells analyzed** | n=9720 | n=8985 | n=15509 | n=26535 |
| **Mean total # of cortical cells/section** | 3240.0 ± 135.6 | 2995.0 ± 489.3 | 5169.7 ± 107.1 | 8845.0 ± 2605.2 |
| **Mean # of TBR2+ cells/section** | 660.3 ± 51.4 | 1007.3 ± 139.5 | 1284.0 ± 117.2 | 378.3 ± 57.0 |
| **% of TBR2+ cells** | 20.3 ± 1.0 | 33.9 ± 1.4 | 24.9 ± 2.4 | 5.3 ± 1.9 |
| **Mean ventricular surface length (μm)** | 960.0 ± 31.1 | 860.7 ± 42.3 | 1286.2 ± 44.6 | 2835.2 ± 466.1 |
| **Mean # of TBR2+ cells/mm ventricle** | 687.3 ± 45.3 | 1164.6 ± 121.9 | 999.24 ± 95.9 | 149.0 ± 47.3 |
| **Basal PH3+ cells/mm ventricle** | 28.7 ± 2.0 | 59.2 ± 5.4 | 32.4 ± 4.8 | 17.1 ± 3.4 |

**Table S1. Summary of experimental data.**

All values are ± s.e.m.
